# Prescription Department

**Published:** 1897-12

**Authors:** 


### METHYL SALICYLATE IN THE TREATMENT OF GONORRHEA.

By reason of the great power possessed by methyl salicylate of penetrating investing membrane, M. Duquaire (cited in the *Journal des Practiciens* for June 26th) has conceived the idea that it will reach the gonococci even when they are seated in the deepest layers of the mucous membrane. He reports a case in which its employment cured the disease in five days, but he does not generalize from that one case. He usesthe following solution:

℞. Methyl salicylate... .....1 part.
Bismuth subnitrate.... .20 parts.
Liquid vaseline.......100 parts.

M. These injections are to be given daily. The patient should urinate, then take the injection, and hold it in the urethra as long as possible. The injections are not painful.—*New York Medical Journal.*

### MALARIA IN CHILDREN.

A. Zuckerman uses the following:

℞. Tinct. eucalypt. glob.
Spts. vini. dilut........aa 8.0
Quin. mur ... ........ 2.0
Chinoidini ..... .....1.25 to 2.0
Acid mur. dil., q. s. ad. solut.

M. Sig: 20 to 40 drops five times a day.

—Esheledelnik russ, 1895, 34

